# Bariatric Surgery in Morbidly Obese Adolescents: a Systematic Review and Meta-analysis

**DOI:** 10.1007/s11695-015-1581-2

**Published:** 2015-02-20

**Authors:** Givan F. Paulus, Loes E. G. de Vaan, Froukje J. Verdam, Nicole D. Bouvy, Ton A. W. Ambergen, L. W. Ernest van Heurn

**Affiliations:** 1 Department of Surgery, Maastricht University Medical Center, and Nutrition and Toxicology Research Institute (NUTRIM), P. Debyelaan 25, 6229 HX Maastricht, The Netherlands; 2 Department of Methodology and Statistics, Maastricht University, Maastricht, The Netherlands; 3 Department of Otolaryngology, University Medical Centre Utrecht, Utrecht, The Netherlands

**Keywords:** Adolescents, RYGB, LAGB, LSG, Bariatric surgery, Meta-analysis, Review, Weight loss, Quality of life

## Abstract

Pubmed, Embase, and Cochrane were systematically reviewed for available evidence on bariatric surgery in adolescents. Thirty-seven included studies evaluated the effect of laparoscopic adjustable gastric banding (LAGB), Roux-en-Y gastric bypass (RYGB), or laparoscopic sleeve gastrectomy (LSG) in patients ≤18 years old. Fifteen of 37 studies were prospective, including one RCT. Mean body mass index (BMI) loss after LAGB was 11.6 kg/m^2^ (95 % CI 9.8–13.4), versus 16.6 kg/m^2^ (95 % CI 13.4–19.8) after RYGB and 14.1 kg/m^2^ (95 % CI 10.8–17.5) after LSG. Two unrelated deaths were reported after 495 RYGB procedures. All three bariatric procedures result in substantial weight loss and improvement of comorbidity with an acceptable complication rate, indicating that surgical intervention is applicable in appropriately selected morbidly obese adolescents.

## Introduction

Obesity is an emerging pandemic phenomenon [1]. Over the past three decades, the prevalence of adult obesity in the USA has doubled, while that of adolescent obesity has tripled [2]. Current estimates classify 33.6 % of adolescents living in the USA as overweight, 18.4 % as obese, and 13.0 % as being extremely obese, defined as body mass index (BMI) ≥85th, 95th, and 97th percentile, respectively [3]. Individual, social, environmental, and economic factors contribute to the development and persistence of morbid obesity.

Adolescent obesity is associated with preventable chronic health conditions like type two diabetes mellitus (T2DM), hypertension, obstructive sleep apnea syndrome (OSAS), dyslipidemia, nonalcoholic steatohepatitis, polycystic ovary syndrome, and various musculoskeletal diseases [4, 5]. Obese adolescents are likely to suffer from psychological morbidity, loss of self-esteem, and social exclusion which has the potential to scar them for life [6]. The risk of dying from any obesity-related cause increases by 6–7 % for every 2 years lived with obesity [7]. These findings urge us to find ways to treat obesity early in life.

Presently, adolescent obesity is mostly managed by combined lifestyle interventions focusing on behavioral and dietary modifications. These treatments are typically initiated and evaluated by a multidisciplinary team including a pediatrician, dietician, psychologist, and a physiotherapist. While often effective in short term, long-term effects are relatively disappointing. A recent Cochrane review shows a maximum of 1.7 kg/m^2^ BMI loss after 12 months of lifestyle intervention [8].

In adults, bariatric surgery is extremely effective compared to conservative treatment, resulting in adequate long-term weight loss and reduction of mortality [9]. The last decades, various bariatric procedures have been performed in adolescents, including laparoscopic adjustable gastric banding (LAGB), Roux-en-Y gastric bypass (RYGB), vertical banded gastroplasty, biliopancreatic diversion, and more recently laparoscopic sleeve gastrectomy (LSG). Potential adverse effects on growth and development in prepubertal patients who have not reached full maturity raise concerns. However, bariatric surgery relatively early in life intervenes before comorbidities become irreversible and reduces the risk of surgical complications.

Currently, the guidelines from the International Pediatric Endosurgery Group (IPEG) state that adolescents with a BMI >40 kg/m^2^ or a BMI >35 kg/m^2^ combined with severe comorbidities should be considered for surgical intervention, if they have (nearly) attained adult stature [10]. These guidelines are largely based upon a systematic review and meta-analysis by Treadwell et al. [11], reviewing studies up to December 2007. The last few years, indication criteria for bariatric surgery have expanded, and surgical techniques have improved. However, the outcome and best techniques to treat morbidly obese adolescents remain relatively unknown.

In this review, we evaluate and compare the efficacy, safety, and (psychosocial) health benefits of various bariatric surgical techniques as a treatment for morbid obesity in adolescents. Our data are obtained with help of supplemental data from several authors and strengthened by inclusion of the most recent high-quality studies.

## Methods

### Protocol and Registration

This review was conducted according to the PRISMA [12] and MOOSE [13] statements.

### Eligibility Criteria

Prospective clinical trials and observational studies on LAGB, RYGB, and LSG were included with the following inclusion criteria: ≥10 patients, mean follow-up ≥12 months, age ≤18 years at time of operation (and less than 20 % >18 years), majority of procedures <25 years ago, and English full-text available. Meta-analysis of BMI loss was done when BMI loss was either reported or could be calculated.

### Search

**Fig. 1:**
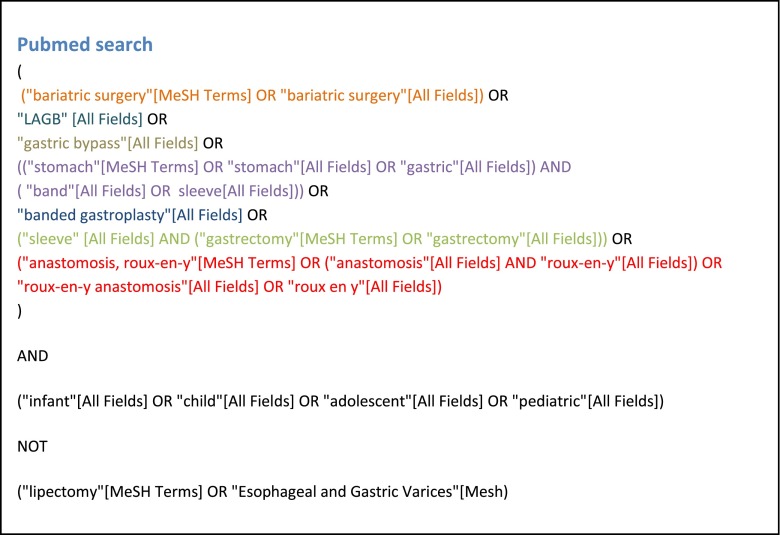
Search terms: full Pubmed search

Pubmed, Embase, and Cochrane databases were searched on the 20 January 2014 with relevant search terms and Medical Subject Headings (MeSH) on LAGB, RYGB, and LSG in children and adolescents. Full electronic Pubmed search is presented in Fig. 1.

### Study Selection

After electronically removing duplicates using EndNote X6.0.1 (Thomson Reuters), all remaining duplicate entries and aberrant records were manually removed. Two independent researchers (GP and LdV) screened the remaining abstracts and/or full-text version and collected the eligible citations. Clinical data and study properties were added to the citations by reviewing all full-text articles. Reviewing inclusion period, surgical center, authors, and population characteristics identified publications with data overlap; in which case, articles presenting the most complete and/or recent data were included.

### Data Collection Process

Data relevant for our systematic review and meta-analysis were collected in a datasheet and completed with data from referenced articles or previous publications or by contacting the corresponding author.

### Data Items

BMI before and after the procedure or BMI loss with reported variance, complications, and change in comorbidity was extracted from each article. When individual patient data were available, mean BMI and variance were calculated for those patients younger than 19 years. Mean BMI at follow-up was only used to calculate BMI loss if more than 50 % of the baseline population had reached that moment.

### Risk of Bias in Individual Studies

Study characteristics that influence risk of bias (e.g. prospective/retrospective) were assessed and collected in a table. Additionally, two independent reviewers carefully assessed details on the in- and exclusion process, preoperative lifestyle treatment, postoperative lifestyle support and loss to follow-up.

### Summary Measures

Mean BMI loss was used for meta-analysis. Corresponding authors were contacted if variance of BMI loss was not reported. Complications and comorbidity resolution were summarized if follow-up was at least 6 months. Minor complications, reported in less than three studies, were omitted from the results.

### Synthesis of Results

Summary effect measure of BMI loss and forest plots were produced with 95 % CI for each surgical method using STATA (StataCorp. 2013. *Stata Statistical Software: Release 13*. College Station, TX, USA). Differences between operative techniques were tested in a random effect model. For missing variances, the square root of the average sample-size-weighted variance from all available variances was used. Data on complications or comorbidities were summarized when they were specifically mentioned. Results from large multicenter database studies were not summarized, while for short-term studies (<6-month follow-up), only perioperative results were summarized.

### Risk of Bias Across Studies

A funnel plot for standard error of BMI loss against BMI loss was used to assess publication bias for each technique. The straight lines indicate the region within which 95 % of points should lie in the absence of both heterogeneity and publication bias (Fig. 4).

### Additional Analyses

A meta-regression analysis was performed to assess if BMI loss was affected by follow-up duration after the first 12 months or by different surgical gastric banding techniques (perigastric vs. pars flaccida). Authors were contacted when technical details were not provided. Additionally, differences in baseline BMI of different surgical procedures were tested in a random effect model.

## Results

### Study Selection

**Fig. 2:**
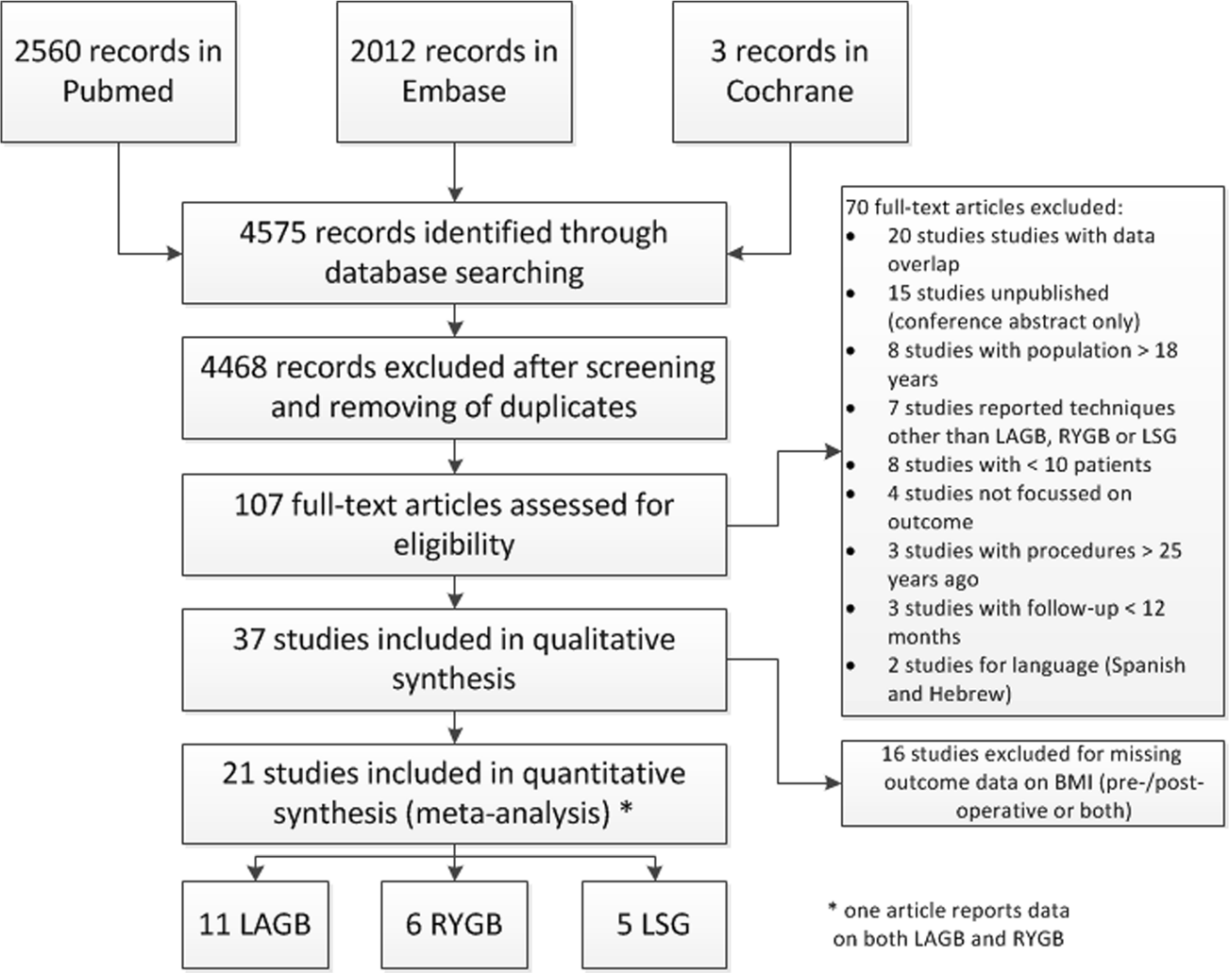
Search diagram: paper retrieval schematic

The search in Pubmed, Embase, and Cochrane provided a total of 4575 citations. After removing duplicates and screening abstracts, 4468 records were excluded and 107 remained for full-text analysis. Seventy full-text articles did not meet the inclusion criteria. Therefore, a total of 37 articles were included, including one article reporting on both LAGB and LSG. Eleven of 18 LAGB studies, 6 of 13 RYGB studies, and 5 of 7 LSG studies were eligible for meta-analysis of BMI loss (Table 1, Fig. 2). No additional studies were identified through cross-referencing.

**Table 1:** Study characteristics

Authors	Operation period	Location	N	Follow-up (months)	Age (years; mean/range)	Operative technique details	Design	Included for
Studies on LAGB								
Abu-Abeid et al. [27]	NR	Tel-Aviv, Israel	11	6–36	15.7	Perigastric	Retrospective	M – CO – CM
Al-Qahtani [28]	Jan, 2003–12/2005	Riyadh, Saudi Arabia	51	3–34	16.8	Pars flaccida	Retrospective	CM
Alqahtani [29]	6/2004–12/2007	Riyadh, Saudi Arabia	50	NR–60	17	Pars flaccida	Retrospective	CO
Angrisani et al. [30]	1/1996–12/2003	Naples, Italy	58	0–84	18.0	55 perigastric; 3 pars flaccida	Retrospective	M – CO – CM
Dolan et al. [31]	1996–NR	Brisbane, Australia—Royal Brisbane Hospital	17	12–46	16.7	Since 1999 pars flaccida	Prospective	M
Fielding et al. [32]	1998–2003	Brisbane, Australia—Wesley Hospital	41	1–70	15.6	Since 1999 pars flaccida	Retrospective	CO – CM
Holterman et al. [14]	3/2005–6/2007	Chicago, IL, USA	20	15–42	16	Perigastric	Prospective	M – CO – CM – QOL
Inge et al. [33]	2/2007–12/2011	Five centers, USA	14	1	17.1	Pars flaccida	Prospective	CO
Lee et al. [34]	2002–2011	New York, NY, USA—St. Luke’s-Roosevelt Hospital Center	23	1–24	17.2	Pars flaccida	Retrospective	CO – CM
Lennerz et al. [35]	1/2005–12/2010	23 centers, Germany	10	0 to >30	16.7	NA	Prospective	M
Messiah et al. [36]	4/2004–10/2010	360 facilities, USA	436	0–12	18.5	NA	Prospective database	CM
Nadler et al. [37]	9/2001–1/2007	New York, NY, USA—NY University School of Medicine	73	12–24	15.8	Pars flaccida	Prospective	M – CO – CM
O’Brien et al. [15]	5/2005–9/2008	Melbourne, Australia	25	24	16.5	Pars flaccida	RCT	M – CO – QOL
Silberhumer et al. [17]	1998–2004	Salzburg/Vienna, Austria	50	63–138	17.1	Pars flaccida	Retrospective multicenter	M – CO – CM – QOL
Silva et al. [38]	7/2001–6/2010	Oporto, Portugal	14	12–36	16.3	Pars flaccida		M – CO – CM
Varela et al. [39]	2002–2006	59 university centers, USA	90	1	12–18	NA	Retrospective	CO
Yitzhak et al. [18]	2000–2003	Beer Sheva, Israel	60	25–65	16	Two pars flaccida techniques	Retrospective	M – CO – CM – QOL
Zitsman et al. [40]	8/2006–NR	New York, NY, USA—Columbia University Medical Center	100	12	14–19	Pars flaccida	NR	M – CO – CM
Studies on RYGB								
De la Cruz-Munoz et al. [41]	2001–2010	Miami, FL, USA	71	9–15	18.3	NR	Retrospective	M – CO
Inge et al. [42]	2001–2003	Cincinnati, OH, USA	10	1–24	NR	Two open/Eight laparoscopic, hand-sewn gastrojejunostomy	Retrospective	CO – CM
Inge et al. [33]	2/2007–12/2011	Five centers, USA	161	1	17.1	NA	Prospective	CO
Lee et al. [34]	2002–2011	New York, NY, USA—St. Luke’s-Roosevelt Hospital Center	32	1–24	18.6	Pouch 50 mL/40-cm biliopancreatic limb, 100-cm alimentary limb	Retrospective	CO – CM
Messiah et al. [36]	4/2004–10/2010	360 facilities, USA	454	12	18.5	NA	Prospective database	CM
Miyano et al. [43]	8/2002–5/2007	Cincinnati, OH, USA	77	3	16.8	Biliopancreatic limb 75–150 cm/15–30 cm from Treitz/30–45-mL pouch	Retrospective	CO – CM
Nijhawan et al. [44]	2001–2007 (approx.)	San Diego, CA, USA	20	60–120	16.9	Pouch 15 mL/Roux limb 75 cm	Retrospective	M – CO – CM
Olbers et al. [22]	2/2006–6/2009	Gothenburg, Sweden	81	24	16.5	Pouch <20 mL/Roux limb 80 cm	Prospective	M – CO – CM – QOL
Strauss et al. [45]	4/1985–5/1999	New Brunswick, NJ, USA	10	8–156	16.2	Pouch 20 ± 5 mL/Roux limb 50–150 cm or to distal jejunum	Retrospective	M – CO – CM
Sugerman et al. [19]	1981–1/2002	Richmond, VA, USA	33	1–14	16	Standard, long-limb, and distal gastric bypass	Retrospective	M – CO – CM
Varela et al. [39]	2002–2006	59 university centers, USA	191	1	12-18	NA	Retrospective	CO
Zeller et al. [20]	5/2004–1/2007	Cincinnati, OH, USA	31	12	16.4	Pouch 20 mL/5–10 cm from Treitz/Roux limb 100–150 cm [46]	Prospective	QOL
Zeller et al. [21]	5/2004–9/2005	Cincinnati, OH, USA	16/14	24	16.2	Pouch 20 mL/5–10 cm from Treitz/Roux limb 100–150 cm [46]	Prospective	M – QOL
Studies on LSG								
Aldaqal et al. [23]	11/2009–2/2012	Jeddah, Saudi Arabia	32	12	15.2	50–80-mL lumen	Prospective	M – CO – CM – QOL
Alqahtani et al. [47]	3/2006–2/2011	Riyadh, Saudi Arabia	99	6–24	14	NR	Retrospective	M – CO – CM
Boza et al. [48]	1/2006–10/2009	Santiago, Chile	51	6–24	18	60-F calibration catheter	Retrospective	M – CO – CM
Inge et al. [33]	2/2007–12/2011	Five centers, USA	67	1	17.1	NA	Prospective	CO
Lennerz et al. [35]	1/2005–12/2010	23 centers, Germany	11	12	15.4	NA	Prospective	M – CM
Nadler et al. [49]	1/2010–12/2011	Washington, DC, USA	23	9–15	17.3	40-F bougie	Retrospective	M – CO – CM
Varela et al. [39]	2002–2006	59 university centers, USA	28	1	12–18	NA	Retrospective	CO

Studies included for meta-analysis and systematic review, marked gray if only eligible for semiquantitative analysis

*NR* not reported, *NA* not applicable, *M* meta-analysis, *CO* complications, *CM* comorbidity, *QOL* quality of life assessment

### Risk of Bias Within Studies

The study design (randomized control trial (RCT), prospective, and retrospective) and study characteristics are presented in Table 1. Potential introducers of bias, other than design, are reported in Table 2. Of 18 LAGB studies, seven were prospective, including the only RCT in this review. Five of 13 RYGB studies were prospective and three of seven LSG studies.

**Table 2:** Risk of bias: list of factors that introduce a risk of bias

Study	Inclusion and exclusion criteria	Intervention before surgery	Support after surgery	Loss to follow-up
Gastric banding				
Abu-Abeid et al. [27]	NIH criteria	≥1-year dietician	Emotional support	NR
Al-Qahtani [28]	NIH criteria	Failure to lose weight for ≥6 months with conservative treatment	Flexible follow-up, reinforcement of the importance of diet and exercise	NR
Alqahtani [29]	NIH criteria	Failure to lose weight for ≥6 months with conservative treatment	Flexible follow-up, reinforcement of the importance of diet and exercise	NR
Angrisani et al. [30]	BMI ≥40 or ≥35 kg/m^2^ with comorbidities, psychiatric and genetic disorders excluded	≥1 year of conservative medical treatment	NR	8–12–24 % [12–36–60 months)
De la Cruz-Munoz et al. [41]	NIH criteria	NR	NR	9 % for LAGB + RYGB
Dolan et al. [31]	NR (2/17 patients BMI < 35)	NR	NR	0–31 % (12–24 months)
Fielding et al. [32]	BMI ≥40 or ≥35 kg/m^2^ with comorbidities	“Appropriate pediatric care”	Surgeon alone	2 %
Holterman et al. [14]	NIH criteria	4–6-month multidisciplinary program	Behavioral, nutritional, and activity monitoring and monthly counseling	20 %
Inge et al. [33]	Pratt [50], BMI ≥35 with major comorbidities and BMI ≥40 with other comorbidities, no binge-purge eating disorders	NR	NR	NR
Lee et al. [34]	NIH criteria, procedure choice on individual basis	Exercise and diet with nutritionist, educational sessions, and psychological and nutritional evaluations	NR	70 % (24 months)
Lennerz et al. [35]	CAADIP 2010 and IFSO guidelines, procedure choice on individual basis	NR	Multidisciplinary approach including a pediatrician, child psychologist, surgeon, and the primary care provider	53 % (LAGB + LSG)
Messiah et al. [36]	NA (national database)	NA	NA	12–3–63 % (3–6–12 months)
Nadler et al. [37]	NIH criteria	NR	First postoperative year monthly to monitor weight loss, appetite, dysphagia, or food intolerance and eating behavior; every 3 months after the first year	11 %
O’Brien et al. [15]	BMI >35 kg/m^2^, identifiable medical complications, physical limitations, or psychosocial difficulties	>3 years of attempts to lose weight by lifestyle means	Participants were encouraged to do exercise and maintain a high level of activity	4 %
Silberhumer et al. [17]	>99.5th age- and gender-adjusted growing percentile, adolescents <14 years old at least one comorbidity	Diet camps, behavioral therapy, and drug therapy	3, 6, and 12 months after surgery by a surgeon; pediatricians and psychologists on a regular basis	10 %
Silva et al. [38]	IPEG guidelines	NR	NR	0 %
Varela et al. [39]	NA (national database)	NA	NA	NA
Yitzhak et al. [18]	NIH criteria	Failed conservative treatment	NR	0 %
Zitsman et al. [40]	Pratt [50], BMI ≥35 with major comorbidities and BMI ≥40 with other comorbidities, no binge-purge eating disorders	NR	Follow-up visits, no support program	0 %
Gastric bypass				
De la Cruz-Munoz et al. [41]	NIH criteria	NR	NR	9 % for LAGB + RYGB
Inge et al. [42]	BMI ≥40 kg/m^2^ with serious obesity-related comorbidities or BMI ≥50 kg/m^2^ with other comorbidities	≥6 months of organized attempts at weight management	Regular visits with the surgeon, psychologist, and dietician	NR
Inge et al. [33]	Pratt [50], BMI ≥35 with major comorbidities and BMI ≥40 with other comorbidities; no binge-purge eating disorders	NR	NR	NR
Lee et al. [34]	NIH criteria, procedure choice on individual basis	Exercise and diet with nutritionist, educational sessions, and psychological and nutritional evaluations	NR	84 % (24 months)
Messiah et al. [36]	NA (national database)	NA	NA	12–34–63 % (3–6–12 months)
Miyano et al. [51]	2002–2006, BMI ≥40 kg/m^2^ with serious obesity-related comorbidities or BMI ≥ 50 kg/m^2^ with other comorbidities 2006–2007, BMI ≥35 kg/m^2^ with serious obesity-related comorbidities or BMI ≥40 kg/m^2^ with other comorbidities	≥6 months of organized attempts at weight management	Regular visits with the surgeon, psychologist, and dietician	NR
Nijhawan et al. [44]	NR	NR	Follow-up visits, encourage support groups	20 %
Olbers et al. [22]	BMI >40 or BMI >35 kg/m^2^ with comorbidity, pubertal Tanner stage > III and passed peak height growth velocity, no untreated psychiatric disorder	Multidisciplinary lifestyle intervention	Follow-up visits, no support program	0 %
Strauss et al. [45]	NR	Serious attempts at weight loss in diet and behavior modification programs	NR	10 %
Sugerman et al. [19]	NIH criteria	NR	NR	3.1–6.7–22.2–33.3 % (1–5–10–14 years)
Varela et al. [39]	NA (national database)	NA	NA	NA
Zeller et al. [20]	Inge: BMI ≥40 with comorbidity or ≥50 [52]	Inge, ≥6 months of organized attempts at weight management	NR	10 % (12 months)
Zeller et al. [21]	Inge: BMI ≥40 with comorbidity or ≥50 [52]	Inge, ≥6 months of organized attempts at weight management	NR	12 %
Sleeve gastrectomy				
Aldaqal et al. [23]	BMI ≥40 kg/m^2^ with serious obesity-related comorbidities or BMI ≥50 kg/m^2^ with other comorbidities	>6 months of recognized, medically supervised weight loss attempts	NR	NR
Alqahtani et al. [47]	BMI ≥40 or ≥35 kg/m^2^ with comorbidities (five patients with BMI <35)	6 months in a formal weight loss program	Follow-up visits	17–14 % (12–24 months)
Boza et al. [48]	NIH criteria, evaluation by multidisciplinary team	NR	NR	13–17 % (12–24 months)
Inge et al. [33]	Pratt [50], BMI ≥35 with major comorbidities and BMI ≥40 with other comorbidities, no binge-purge eating disorders	NR	NR	NR
Lee et al. [34]	NIH criteria, procedure choice on individual basis	Exercise and diet with nutritionist, educational sessions, and psychological evaluations	NR	70 % (24 months)
Lennertz et al. [35]	CAADIP 2010 and IFSO guidelines, procedure choice on individual basis	NR	Multidisciplinary approach including a pediatrician, child psychologist, surgeon, and the primary care provider	53 % (LSG + LAGB)
Nadler et al. [49]	NIH criteria	NR	Follow-up visits, no program	19–0 % (6–12 months)
Oberbach et al. [53]	Inge: BMI ≥40 with comorbidity or ≥50 [52]	“Every conservative treatment had failed”	NR	NR
Varela et al. [39]	NA (national database)	NA	NA	NA

NIH, CAADIP, IFSO criteria, BMI ≥40 kg/m^2^ or BMI ≥35 kg/m^2^ with associated comorbidities [54–56]; IPEG guideline, BMI ≥35 kg/m^2^ with severe comorbidities or BMI ≥40 kg/m^2^ with other comorbidity [10]

*NA* not applicable, *NR* not reported

### Results of Individual Studies

In 15 of the 22 included datasets, SD of BMI loss was not reported or available. Nine of the contacted research groups were willing to supply data on BMI loss with SD at one or more follow-up moments to complete the dataset. Finally, 14 SDs were available and 8 were derived as stated in the methods.

### Synthesis of Results

**Fig. 3:**
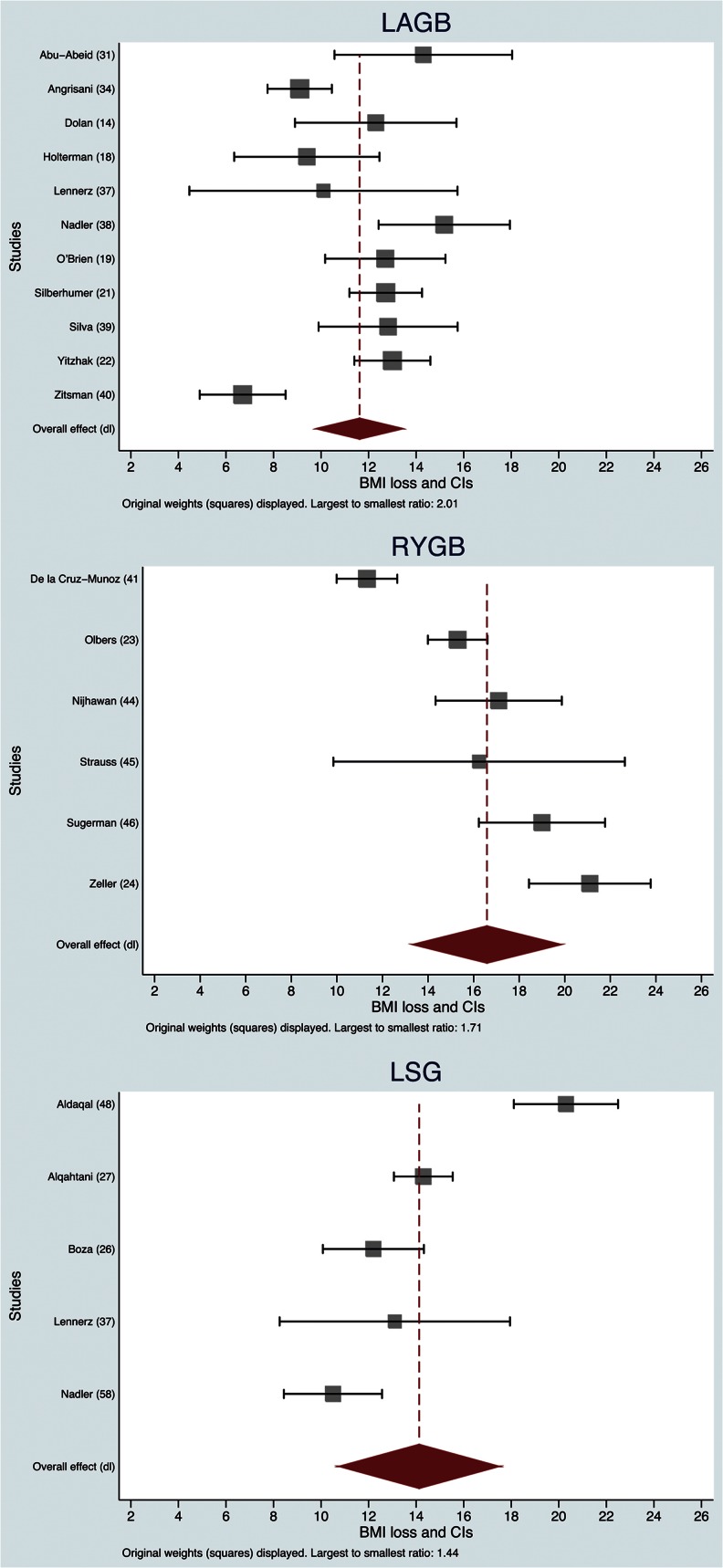
Meta-analysis: forest plot for BMI loss with 95 % confidence intervals and summarized means after LAGB, RYGB, and LSG

Per procedure, a short summary is provided of weight loss, complications, comorbidity reduction, and quality of life assessment (QOL). An overview is provided in Tables 3, 4, and 5 and in Fig. 3.

**Table 3:** BMI loss data used for meta-analysis

Study	N (at FU)	FU (months)	BMI baseline	SD	BMI loss	SD
Gastric banding						
Perigastric technique						
Abu-Abeid [27]	11	23	46.4	NR	14.3^a^	NR
Angrisani [30]	37	36	46.1	6.31	9.1^b^	4.2
Dolan [31]	9	24	42.6	6.7	12.3^a^	5.2
Holterman [14]	12	18	50	10	9.4^a^	5.4
Pars flaccida technique						
Lennerz [35]	10	12	48.1	9.8	10.1^a^	9.1
Nadler [37]	47	12	47.6	7	15.2^b^	9.7
O’Brien [15]	24	24	42.3	6.1	12.7^a^	NR
Silberhumer [17]	48	36	45.2	7.6	12.7^b^	5.4
Silva [38]	12	36	46.1	11.8	12.8^b^	5.2
Yitzhak [18]	60	39.5	43	NR	13^a^	NR
Zitsman [40]	47	12	50 (M) 48.1 (F)	NR	6.7^a^	NR
Gastric bypass						
De la Cruz-Munoz [41]	71	9-15	46.2	5.1	11.3^b^	5.7
Nijhawan [44]	20	85.8	45.7	NR	17.1^a^	NR
Olbers [22]	81	24	45.5	6.0	15.3^b^	6.0
Strauss [45]	10	68.8	52.4	10.1	16.2^c^	10.3
Sugerman [19]	20	60	52	11	19^a^	NR
Zeller [20]	14	24	59.9	8.7	21.1^b^	5.1
Sleeve gastrectomy						
Aldaqal [23]	32	12	49.6	4.9	20.3	NR
Alqahtani [47]	76	6	49.6 (median)	11.5 (IQR)	14.3^b^	5.5
Boza [48]	34	24	38.5	3.7	12.2^a^	NR
Lennerz [35]	11	12	51.8	8.3	13.1^a^	8.2
Nadler [57]	13	6	52	9	10.5^b^	3.8

Male (M), female (F)

^a^From manuscript

^b^From author

^c^Calculated from individual data

**Table 4:** Complications

Authors	N	FU	Complication										Total	Intervention									Total
			Death	Perioperative complications	Surgical site infection	Late complications	Hiatal hernia	Band-specific	Gastrointestinal complaints	Nutritional deficiency / dehydration	DVT	Pulmonary system (pneumonia, pulmonary embolism)		Conversion to malabsorptive anatomy	Band, removal	Band replacement/repositioning	Band, port revision	Gastrointestinal obstruction	Leak/fistula repair	Cholecystectomy	Abdominal hernia repair	EGD	
LAGB																							
Perigastric																							
Abu-Abeid et al.	11	6–36 months	*	0	*	0	*	0	0	*	*	*	0	*	*	*	*	*	*	*	*	*	0
Angrisani et al.	58	0–7 years	0	1	*	*	*	6	*	*	*	*	7	3	5	3	*	*	*	*	*	*	11
Holterman et al.	20	15–42 months	*	*	*	*	1	4	*	*	*	*	5	*	*	1	3	*	*	*	1	*	5
Pars flaccida																							
Alqahtani	50	NR–5 years	*	0	*	*	*	2	9	1	*	*	12	*	2	*	*	*	*	*	0	*	2
Fielding et al.	41	1–70 months	0	0	*	*	*	2	*	*	*	*	2	*	*	1	1	*	*	*	*	*	2
Lee et al.	23	1–24 months	*	*	*	*	*	2	*	*	*	*	2	*	1	1	*	*	*	*	*	*	2
Nadler et al.	73	12–24 months	0	*	1	1	3	7	5	*	*	*	17	*	2	5	1	*	*	1	3	*	12
O’Brien et al.	24	24 months	*	0	*	*	*	8	1	*	*	*	9	*	*	6	2	*	*	1	*	*	9
Silberhumer et al.	50	63–138 months	*	*	*	*	*	6	*	*	*	*	6	8	*	2	2	*	*	*	*	*	12
Silva et al.	14	12–36 months	0	0	*	*	*	2	2	*	*	*	4	*	*	*	2	*	*	1	*	*	3
Yitzhak et al.	60	25–65 months	0	0	*	*	*	10	*	*	*	*	10	*	2	6	2	*	*	*	*	*	10
Zitsman et al.	100	12 months	0	1	*	1	1	6	*	*	*	*	9	*	*	3	3	2	*	*	1	*	9
Inge et al.	14	30 days	0†	1	0†	0†	*†	*†	1†	*†	0†	1†	3†	*	*	*	*	0	*	*	0	*	0†
TOTAL	538		0 % (0/346)	0.8 % (3/372)	1.4 % (1/73)	1.1 % (2/184)	2.6 % (5/193)	10.5 % (55/524)	9.9 % (17/172)	2 % (1/50)	*	*	83	11	12	28	16	2	0	3	5	0	14.7 % (77/524)
Short-term perioperative outcome																							
Varela et al.	90	30 days	0	*	*	*	*	*	*	*	*	*	0	*	*	*	*	*	*	*	*	*	0
RYGB																							
De la Cruz-Munoz et al.	71	9–1 months	*	0	*	*	*	*	2	*	*	*	2	*	*	*	*	*	*	*	*	*	0
Inge et al.	10	1 month–2 years	*	1	*	3	*	*	1	1	1	*	7	*	*	*	*	*	1	*	*	1	2
Lee et al.	32	1–24 months	*	*	*	1	*	*	*	1	*	*	2	*	*	*	*	*	1	*	*	*	1
Miyano et al.	77	90 days	0	2	2	24	*	*	*	5	1	0	34	*	*	*	*	4	4	*	2	13	23
Nijhawan et al.	20	60–120 months	0	0	1	3	*	*	*	*	*	1	5	*	*	*	*	2	0	*	*	1	3
Olbers et al.	81	24 months	0	2	*	6	*	*	11	*	*	*	19	*	*	*	*	5	0	5	2	*	12
Strauss et al.	10	8–156 months	*	0	*	2	*	*	2	1	*	*	5	*	*	*	*	1	*	2	1	*	4
Sugerman et al.	33	1–14 years	2	*	5	14	*	*	*	1	*	1	22	2	*	*	*	1	*	*	6	3	12
Inge et al.	161	30 days	0†	17	3†	9†	*†	*†	*†	*†	1†	2†	17†	*	*	*	*	3	4	*	*	3	10†
TOTAL	495		0.9 % (2/211)	5.1 % (22/430)	6.2 % (8/130)	20.2 % (53/263)	*	*	9.3 % (16/172)	5.6 % (9/162)	2.3 % (2/87)	1.5 % (2/130)	96	2	0	0	0	13	6	7	11	18	17.1 % (57/334)
Short-term perioperative outcome																							
Varela et al.	191	30 days	0	*	*	*	*	*	*	*	*	*	4.3–7.6 %	*	*	*	*	*	*	*	*	*	0
LSG																							
Aldaqal et al.	32	12 months	*	0	*	*	*	*	*	*	*	*	0	*	*	*	*	*	*	*	*	*	0
Alqahtani et al.	99	6–24 months	0	0	2	1	*	*	3	*	*	*	6	*	*	*	*	*	*	*	*	*	0
Boza et al.	51	6–24 months	0	0	*	1	*	*	*	*	*	*	1	*	*	*	*	*	1	*	*	1	2
Nadler et al.	23	9–15 months	*	0	*	0	*	*	3	*	*	*	3	*	*	*	*	*	*	*	*	*	0
Inge et al.	67	30 days	0†	2	2†	3†	*†	*†	2†	1†	0†	1†	11†	*	*	*	*	0	2	*	0	*	2†
TOTAL	272		0 % (0/150)	0.7 % (2/272)	2.0 % (2/99)	1.2 % (2/173)	*	*	4.9 % (6/122)	*	*	*	10	0	0	0	0	0	1	0	0	1	1.0 % (2/205)
Short-term perioperative outcome																							
Varela et al.	28	30 days	0	*	*	*	*	*	*	*	*	*	0	*	*	*	*	*	*	*	*	*	0

Complications: death (all cause), perioperative (conversion, bleeding, or organ laceration), surgical site infection, late complications (obstruction, abscess, internal hernia, leak, or incisional hernia), hiatal hernia, band-specific (port revision, slippage, dilated pouch, and band migration), gastrointestinal complaints (nausea, vomiting, intestinal blood loss, diarrhea, GERD, gallstones, and dumping), nutritional deficiency/dehydration, DVT, and pneumonia/pulmonary embolus

“*”not reported, “†” not summarized due to short follow-up

**Table 5:** Comorbidity prevalence and reduction

Author		HT	Dyslipidemia	T2DM	Prediabetes/Insulin resistance	OSAS	Musculoskeletal complaints	Asthma	Menstrual problems	GERD
LAGB										
Abu-Abeid et al. [27]	Baseline N (%)	NR	2/11 (18.2 %)†, 1/11 (9.1 %)‡	NR	NR	NR	NR	NR	2/11 (18.2 %)‡	NR
	Resolved N (%)	NR	2/2 (100 %)†, 0/1 (0 %)‡	NR	NR	NR	NR	NR	2/2 (100 %)‡	NR
Al-Qahtani et al. [28]	Baseline N (%)	6/51 (11.8 %)	NR	7/51 (13.7 %)	NR	10/51 (19.6 %)	7/51 (13.7 %)†	NR	NR	NR
	Resolved, N (%)	6/6 (100 %)	NR	7/7 (100 %)	NR	10/10 (100 %)	7/7 (100 %)†	NR	NR	NR
Angrisani [30]	Baseline N (%)	8/58 (13.4 %)	6/58 (10.3 %)	8/58 (13.4 %)	NR	10/58 (17.2 %)	12/58 (20.7 %)†	NR	4/58 (69 %)‡	NR
	Resolved N (%)	NR	NR	NR	NR	NR	NR	NR	NR	NR
Fielding et al. [32]	Baseline N (%)	2/41 (4.9 %)	NR	2/41 (4.9 %)	NR	1/41 (2.4 %)	1/41 (2.4 %)‡	NR	NR	NR
	Resolved N (%)	2/2 (100 %)	NR	2/2 (100 %)	NR	1/1 (100 %)	1/1 (100 %)‡	NR	NR	NR
Holterman et al. [14]	Baseline N (%)	9/20 (45 %)	16/20 (80 %)	NR	18/20 (90 %)†	NR	NR	NR	NR	NR
	Resolved N (%)	9/9 (100 %)	11/16 (67 %)	NR	13/18 (72 %)†	NR	NR	NR	NR	NR
Lee et al. [34]	Baseline N (%)	2/23 (9 %)	2/23 (9 %)‡	0/23 (0 %)	NR	3/23 (13 %)	NR	NR	NR	NR
	Resolved N (%)	NR	1/2 (50 %)	0/0	NR	NR	NR	NR	NR	NR
Messiah et al. [36]	Baseline N (%)	80 (18 %)	61 (14 %)	65 (15 %)	NR	80 (18 %)	113 (25 %)¥; 90 (21 %) #	84 (19 %)	50 (11 %) †;45 (10 %) ¥	109 (25 %)
	Improved N (%)	54 %	23 %	59 %	NR	46 %	50 % ¥44 % #	23 %	38 % †31 % ¥	45 %
Nadler et al. [37]	Baseline N (%)	4/21 (19 %)	7/21 (33 %)	NR	5/21 (24 %)◊	4/21 (19 %)	10/21 (48 %)¥, 5/21 (24 %)†	NR	NR	1/ 21 (5 %)
	Resolved N (%)	4/4 (100 %)	3/7 (43 %)	NR	5/5 (100 %)◊	3/4 (75 %)	7/10 (70 %)¥, 3/5 (60 %)†	NR	NR	1/1 (100 %)
Silberhumer et al. [17]	Baseline N (%)	12/50 (24 %)	4/50(8 %)	5/50 (10 %)	NR	NR	8/50 (16 %)§	3/50 (6 %)	NR	1/50 (2 %)
	Resolved, N (%)	11/12 (91.7 %)	4/4 (100 %)	5/5 (100 %)	NR	NR	7/8 (87.5 %)§	3/3 (100 %)	NR	1/1 (100 %)
Silva et al. [38]	Baseline N (%)	13/14 (92 %)	12/14 (85.7 %)	NR	13 /14 (92.8 %)†	NR	NR	NR	NR	NR
	Resolved, N (%)	13/13 (100 %)	8/12 (66.7 %)	NR	13/13 (100 %)†	NR	NR	NR	NR	NR
Yitzhak et al. [18]	Baseline N (%)	3/60 (5 %)	NR	2/60 (33.3 %)	NR	10/60 (16.7 %)	NR	3/60 (%)	NR	NR
	Resolved, N (%)	3/3 (100 %)	NR	2/2 (100 %)	NR	10/10 (100 %)	NR	3/3 (100 %)	NR	NR
Zitsman et al. [40]	Baseline N (%)	35/85 (41.2 %)	49/85 (57.6 %)	NR	NR	NR	NR	28/85 (32.9 %)	26/85 (31 %)†¥	NR
	Resolved, N (%)	8/35 (22.9 %)	24/49 (49 %)	NR	NR	NR	NR	4/28 (14.3 %)	21/26 (81 %)†¥	NR
RYGB										
Miyano et al. [51]	Baseline N (%)	18 (29 %)	38 (62 %)	8 (13 %)	NR	46 (69 %)	NR	11 (21 %)	11 (24 %) ¥	15 (27 %)
	Resolved, N (%)	NR	NR	NR	NR	NR	NR	NR	NR	NR
Inge et al. [42]	Baseline N (%)	NR	NR	1/10 (10 %)	NR	1/10 (10 %)	NR	NR	NR	NR
	Resolved, N (%)	NR	NR	1/1 (100 %)	NR	1/1 (100 %)	NR	NR	NR	NR
Lee et al. [34]	Baseline N (%)	6/32	2/32 (6 %)	3/32 (%)	NR	5/32	NR	NR	NR	NR
	Resolved, N (%)	NR	2/2 (100 %)‡	3/3 (100 %)	NR	NR	NR	NR	NR	NR
Messiah et al. [36]	Baseline N (%)	118 (26 %)	65 (14 %)	67 (15 %)	NR	117 (26 %)	162 (36 %) ¥ 127 (28 %) #	94 (21 %)	85 (18 %) † 41 (9 %) ¥	127 (28 %)
	Improved N (%)	61 %	59 %	79 %	NR	56 %	50 % ¥ 44 % #	40 %	38 % † 31 % ¥	62 %
Nijhawan et al. [44]	Baseline N (%)	3/25 (12 %)	10/25 (40 %)	3/25 (12 %)	NR	4/25 (16 %)	14/25 (56 %)†	6/25 (24 %)	NR	5/25 (20 %)
	Resolved, N (%)	3/3 (100 %)	10/10 (100 %)	3/3 (100 %)	NR	4/4 (100 %)	13/14 (92.9 %)†	6/6 (100 %)	NR	4/5 (80 %)
Olbers et al. [22]	Baseline N (%)	0/81 (0 %)	15/80 (19 %)†; 27/81 (33 %)◊	1/81 (1.2 %)	17/78 (21 %)¥; 55/78 (70 %)‡	0/81 (0 %)	NR	NR	NR	NR
	Resolved, N (%)	N/A	14/15 (93.3 %)†; 15/27 (55.5 %)◊	1/1 (100 %)	13/17 (76.5 %)¥; 53/55 (96 %)‡	N/A	NR	NR	NR	NR
Strauss et al. [45]	Baseline N (%)	3/10 (30 %)	NR	NR	NR	2/10 (20 %)	1/10 (10 %)◊	NR	NR	NR
	Resolved/improved, N (%)	3/3 (100 %)	NR	NR	NR	2/2 (100 %)	1/1 (100 %)◊	NR	NR	NR
Sugerman et al. [19]	Baseline N (%)	11/33 (33 %)	NR	2/33 (6 %)	NR	6/33(18 %)	11/33 (33 %)	NR	NR	5/33 (15 %)
	Resolved, N (%)	9/11 (82 %)	NR	2/2 (100 %)	NR	6/6 (100 %)	4/11 (36 %)	NR	NR	3/5 (60 %)
LSG										
Aldaqal et al. [23]	Baseline N (%)	4/32 (13 %)	NR	5/32 (16 %)	NR	1/32 (3 %)	NR	NR	NR	NR
	Resolved, N (%)	3/4 (75 %)	NR	4/5 (80 %)	NR	1/1 (100 %)	NR	NR	NR	NR
Alqahtani et al. [47]	Baseline N (%)	39/108 (36.1 %)	52/108 (48.1 %)	22/108 (20.4 %)	14/108 (13 %)¥ or ◊	36/108 (33.3 %)	NR	NR	NR	NR
	Resolved, N (%)	27/36 (75 %)	21/30 (70 %)	15/16 (93.8 %)	11/11 (100 %)¥ or ◊	20/22 (90.9 %)	NR	NR	NR	NR
Boza et al. [48]	Baseline N (%)	4/51 (7.8 %)	12/51 (23.5 %)	2/51 (3.9 %)	27/51 (52.9 %)†	NR	3/51 (5.9 %)†	NR	NR	NR
	Resolved, N (%)	4/4 (100 %)	7/12 (58 %)	1/2 (50 %)	26/27 (96.2 %)†	NR	N/A	NR	NR	NR
Nadler et al. [57]	Baseline N (%)	1/7 (14.3 %)	NR	NR	3/7 (%)†	4/7 (57 %)	1/7 (14.3 %)§	1/7 (14.3 %)	1/7 (14.3 %)¥	1/7 (14.3 %)
	Resolved, N (%)	1/1 (100 %)	NR	NR	3/3 (100 %)†	4/4 (100 %)	1/1 (100 %)§	1/1 (100 %)	1/1 (100 %)¥ improved	1/1 (100 %) improved

Dyslipidemia including “†” elevated triglycerides, “‡” elevated total cholesterol, or “◊” elevated LDL

Prediabetes or insulin resistance defined as “†” HOMA insulin resistance, “◊” impaired glucose tolerance, “¥” elevated fasting glucose, or “‡” elevated fasting insulin

Musculoskeletal problems defined as “†” osteoarthropathy, “‡” Perthes disease of the hip, “¥” back pain, “#” musculoskeletal disorder, “§” orthopedic comorbidities/pain, or “◊” compression fracture of vertebrate

Menstrual problems including “†” menstrual irregularity, “‡” amenorrhea, or “¥” polycystic ovary syndrome

*HT* hypertension, *T2DM* type 2 diabetes mellitus, *OSAS* obstructive sleep apnea syndrome, *GERD* gastroesophageal reflux disease

#### Laparoscopic Adjustable Gastric Band

##### Weight Loss

Summary BMI measure at baseline was 45.8 kg/m^2^ (44.0–47.7). The summary effect measure of BMI loss in nine studies was 11.6 kg/m^2^ (9.8–13.4) (Fig. 3). After the first 12 months, there was no association between length of follow-up and excess BMI loss (*β* = 0.06, *p* = 0.51). Clustering datasets by banding technique showed no differences in BMI loss (pars flaccida vs. perigastric, 11.0 vs. 10.1 kg/m^2^, *p* = 0.61).

##### Complications

Thirteen studies report unique data on complications after gastric banding in a total of 538 patients (Table 4). No deaths occurred in any of the studies. Perioperative complications including intra-abdominal bleeding and conversion to laparotomy were reported in 0.8 % and surgical site infection in 1.4 %. Late complications including bowel obstruction and abdominal wall hernia were reported in 1.1 % of cases. During the total follow-up period (0 to 138 months), 10.5 % of subjects experienced band-related complications (55/524) and 9.9 % (17/172) gastrointestinal complaints (nausea, vomiting, GERD, diarrhea, and gallstones). There were 77 reinterventions (14.7 %), including 3 cholecystectomies. The majority were band-related procedures like replacement or repositioning (*n* = 28), removal (*n* = 12), and port-revision (*n* = 16). Vitamin deficiencies were reported in 5 of 18 studies; oral supplements for iron, vitamin D, folic acid, and zinc deficiencies were prescribed in 0.5 to 36 % of patients, but criteria for deficiencies were poorly defined. Only 2 of 18 studies report standard postoperative vitamin supplementation, while 13 do not mention a standard policy.

##### Resolution of Comorbidities

Out of the 18 LAGB studies included in this review, 11 report data on comorbidity resolution (Table 5). The definitions and cutoff values for comorbidities were specified in 5 of 11 studies and varied between studies. Resolution rates for hypertension, reported in nine studies, range from 22.9 to 100 %; six studies showed complete resolution in all patients. Nine studies report prevalence of dyslipidemia in 8 to 86 %, with eight reporting resolution in 0 to 100 % (median 50 %) of all cases. Six out of seven studies that report on diabetes prevalence in 0 to 33 %, all showed 100 % resolution after surgery. Resolution of prediabetes (three studies, prevalence 24–93 %) ranged from 72 to 100 %.

##### Quality of Life

Holterman et al. [14] showed that 75 % of the children had abnormal scores on the Pediatric Quality of Life Inventory (Peds-QL) at baseline, which improved at 12 and 18 months after surgery. The RCT by O’Brien et al. [15] showed improvements in reported physical functioning, general health, self-esteem, family activities, and change in health with the Child Health Questionnaire (CHQ CF-50) after gastric banding, while the lifestyle group improved only in general health perception. Silberhumer et al. [16, 17] found significant improvement after 35 months by using the BAROS and Moorehead-Ardelt Quality of Life questionnaires (both tests are not specifically validated in children) but no further changes between 3 and 5 years after surgery. Yitzhak et al. [18] report 93 % improvement in physical activity and 72 % improvement in social- and self-esteem with non-validated questionnaires.

##### Pars Flaccida Versus Perigastric Technique

The LAGB-related problems including slippage, pouch dilation, and migration—after a follow-up period of 0–7 years—do not appear to occur more in patients who were operated before the surgeons updated their techniques to the currently used pars flaccida technique (11.2 % (10/89) vs. 10.3 % (45/435)).

#### Roux-en-Y Gastric Bypass

##### Weight Loss

The studies reporting on laparoscopic Roux-en-Y gastric bypass have a summary BMI loss of 16.6 kg/m^2^ (13.4–19.8) after 12 to 86 months (Table 3, Fig. 2). A follow-up period exceeding 12 months was not correlated to BMI loss (*β* = 0.04, *p* = 0.51). BMI loss after RYGB was significantly higher than that after LAGB (*p* = 0.008). Mean preoperative BMI was 49.6 kg/m^2^ (46.4–52.7) and did not differ from LAGB (*p* = 0.11).

##### Complications

Nine studies present summarizable complication rates in a total of 495 patients. Two sudden deaths were reported in one study, 2 and 6 years after surgery, respectively, which were probably unrelated to the procedure. However, no autopsies were performed to determine the cause of death [19]. Perioperative complications including anastomotic leakage, bleeding, and conversion occurred in 5.1 % and infection of the surgical site in 6.2 % of patients. Late complications including obstruction, internal herniation, ulcers, and abdominal wall hernia occurred in 20.2 % of patients.

Gastrointestinal complaints like nausea, vomiting, dumping, and GERD were reported in 9.3 %; nine patients in five studies (5.6 %) suffered from nutritional deficiencies or dehydration requiring hospitalization. Less severe vitamin deficiencies were reported in 6 of 13 studies; oral supplements for iron, vitamin A, vitamin B1, vitamin B12, vitamin D, folic acid, and zinc deficiencies were used in an estimated 4–56 % of patients, but criteria for deficiencies and exact numbers were poorly described. In 5 of 13 studies, postoperative vitamin supplementation was standard policy, while in seven no details are provided. The highest percentage of deficiencies occurred in the study in which no supplements were supplied.

Fifty-seven reinterventions (17.1 %) were performed including cholecystectomy in seven, endoscopic procedures (mainly balloon dilation for stricture of the anastomosis) in 18, surgery for gastrointestinal obstruction in 13, and for leak or fistula repair in six.

##### Resolution of Comorbidities

Eight of the 13 studies on RYGB report data on comorbidity resolution and/or improvement (Table 5). The definitions and cutoff values for comorbidities were specified in five of eight studies and varied between studies. The studies reporting on hypertension (*n* = 4) show 61 to 100 % improvement or resolution. Six to 62 % of the subjects had dyslipidemia, resolving in 56 to 100 %. Diabetes resolved in 79 to 100 %, with resolution in all subjects in five out of six studies.

##### Quality of Life

Quality of life, reported in two studies, showed significant improvement in seven of the eight health domains on the Short Form-36 Health Survey (SF-36) at 1-year follow-up and significantly increased quality of life scores after 6 months, but not after 12 (assessed with the Peds-QL and IWQOL-Kids). Depression scores were significantly less, 6 and 12 months after surgery, than before surgery [20–22].

#### Laparoscopic Sleeve Gastrectomy

##### Weight Loss

Five studies present the results of the relatively new LSG technique with a follow-up between 6 and 24 months. BMI before surgery was 48.1 kg/m^2^ (41.8–54.5), which does not differ from LAGB or RYGB patients (*p* = 0.42 and *p* = 0.50, respectively). BMI loss in these studies is 14.1 kg/m^2^ (10.8–17.5) and does not differ from LAGB and RYGB (*p* = 0.17 and *p* = 0.24, respectively).

##### Complications

Five studies including 272 patients reported two perioperative complications (0.7 %) and no mortality. The incidence of wound infection was 2.0 %, and late complications occurred in 1.2 %, gastrointestinal complaints in 4.9 % (Table 4). Postoperative vitamin supplementation was described in one of seven studies; none of the studies report whether deficiencies occurred.

##### Resolution of Comorbidities

In four out of five studies on LSG, comorbidities are reported (Table 5). The definitions and cutoff values for comorbidities were specified in two of four studies and varied between studies. Hypertension resolved in 75–100 %. Dyslipidemia improved, with resolution rates of 58 to 70 %, and diabetes, reported in three studies, resolved in 50 to 93.8 %.

##### Quality of Life

Aldaqal et al. [23] assessed self-esteem and quality of life at baseline and 1 year after LSG with the Rosenberg self-esteem scale (RSE) and the Peds-QL. Patients improved significantly on the RSE and all six scores of the Peds-QL (including the summary score) 1 year after the procedure.

### Risk of Bias Across Studies

**Fig. 4:**
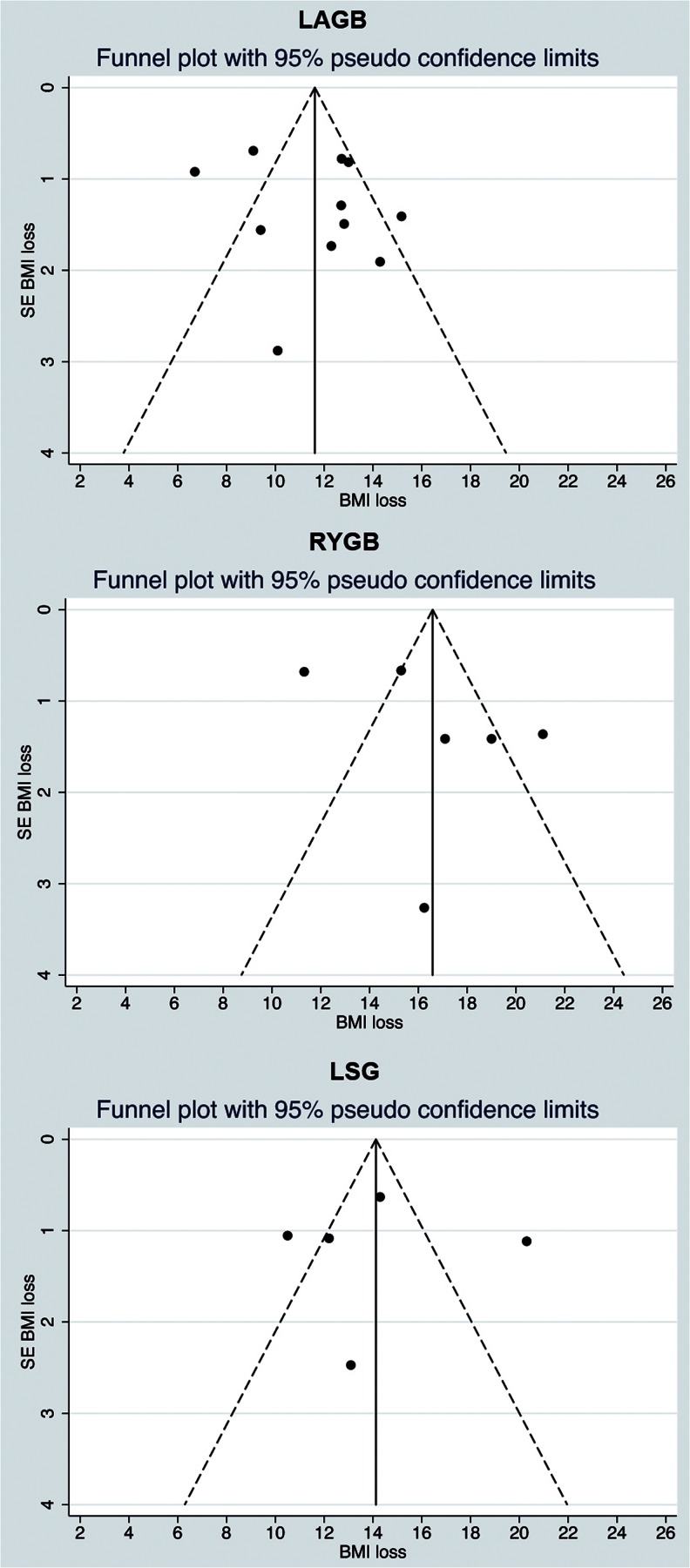
Funnel plots: funnel plots of SEM of BMI loss versus BMI loss for the assessment of heterogeneity in outcome reporting. *Dots outside the 95 % pseudo confidence limits* are indicative of heterogeneity

Figure 4 shows the funnel plots for standard error of BMI loss against BMI loss in each procedure. Eight of the studies reporting on LAGB outcome are within the expected range, while one study shows more and two show less than expected BMI loss. Four RYGB studies are in the expected range, while two are not (one more and one less), and three LSG studies are in the expected range, while two are not (one more and one less).

## Discussion

### Summary of Evidence

The 37 studies that were eligible for systematic reviewing represent the increasing interest in bariatric surgery in morbidly obese adolescents, although the studies were mainly observational and varied in quality. To ensure that the meta-analysis was based on valid data and solidly compares surgical methods, we reported only peer-reviewed published studies and obtained additional data from the authors of nine studies.

All three procedures lead to significant weight loss in morbidly obese adolescents, and similar to a large Swedish study in adults, weight loss is most pronounced after RYGB [9]. This seems to persist after both RYGB and LAGB. For LSG studies, long-term follow-up is not yet available. While adverse events are relatively mild and long-term complication rates are acceptable, they are more frequent and more serious after RYGB than after LAGB. In the currently available follow-up after LSG, the rate of adverse events appears to be similar to that after LAGB. Although a healthy nutritional status in adolescents is important to prevent developmental and growth deficiencies, standard postoperative vitamin supplementation regimens and the occurrence of deficiencies are not reported in most studies (not at all in LSG studies). However, more and more severe deficiencies occur after RYGB than after LAGB.

Reduction of comorbidity, which is pivotal for health gain, is impressive in all techniques, and QOL consistently showed improvement, although follow-up up to 24 months may not be enough to capture negative long-term effects in life after bariatric surgery. The difference in adults between adverse events of the old perigastric LAGB technique and the more recently adapted pars flaccida technique [24] is not reproduced reviewing young patients.

### Limitations

Funnel plots show heterogeneity of the data but no indication of publication bias due to underreporting of poor outcomes. A limitation of the currently available literature is the lack of high-quality, prospective randomized controlled trials, which increases the risk of bias and therefore introduces heterogeneity. Assessment of the three fundamental domains in risk of bias in observational studies (appropriate selection of participants, appropriate measurement of variables, and appropriate control of confounding) shows that studies are heterogeneous in patient selection, in preoperative and postoperative treatment protocol and that loss-to follow-up is substantial. Furthermore, reduction of comorbidity receives sufficient attention in most studies, but varying and lacking definitions of comorbidity introduce another possible source of bias. The similarity in outcome in all studies, however, strengthens our conclusion that the current methods of summarizing BMI loss, complication rate, and reduction of comorbidity are indicative of the true outcome.

## Conclusions

This review is the first that has retrieved sufficient data for meta-analysis of BMI loss by contacting all authors of included studies, to enable a solid statistical analysis. All three analyzed bariatric surgical techniques—laparoscopic adjustable gastric banding, Roux-en-Y gastric bypass, and laparoscopic sleeve gastrectomy—result in substantial weight loss and improvement of comorbidity in the short to medium term. This indicates that, considering the acceptable complication rate, surgical intervention is applicable in appropriately selected adolescents. While BMI loss after RYGB is superior, a higher rate of adverse events and reinterventions has to be taken into account. We recognize that RYGB is currently considered in the treatment of adolescents with a more extreme BMI (>50 kg/m^2^), while LAGB and LSG are applied when obesity is less extreme.

The quality of the available literature is limited. In the current climate where availability of bariatric surgery for morbidly obese children is already increasing, randomized controlled trials comparing bariatric surgery with standard conservative treatment are difficult to perform. Currently, seven active studies are registered in ClinicalTrials.gov assessing the effects of bariatric surgery in adolescents, including one randomized controlled trial. We recommend the involved researchers to use solid outcome reporting strategies and strongly support the pleas for standardized weight loss reporting [25, 26].
